# Transcriptome sequencing of the naked mole rat (*Heterocephalus glaber*) and identification of hypoxia tolerance genes

**DOI:** 10.1242/bio.028548

**Published:** 2017-11-14

**Authors:** Bang Xiao, Li Li, Chang Xu, Shanmin Zhao, Lifang Lin, Jishuai Cheng, Wenjing Yang, Wei Cong, Guanghan Kan, Shufang Cui

**Affiliations:** 1 Laboratory Animal Centre, The Second Military Medical University, Shanghai 200433, China; 2 Department of Training, The Second Military Medical University, Shanghai 20043, China; 3 School of Kinesiology, Shanghai University of Sport, Shanghai 200438, China; 4 China Astronaut Research and Training Center, Beijing 100094, China

**Keywords:** Naked mole rat, Hypoxic stress tolerance, Global transcriptome analysis, Mitogen-activated protein kinase signaling pathway

## Abstract

The naked mole rat (NMR; *Heterocephalus glaber*) is a small rodent species found in regions of Kenya, Ethiopia and Somalia. It has a high tolerance for hypoxia and is thus considered one of the most important natural models for studying hypoxia tolerance mechanisms. The various mechanisms underlying the NMR's hypoxia tolerance are beginning to be understood at different levels of organization, and next-generation sequencing methods promise to expand this understanding to the level of gene expression. In this study, we examined the sequence and transcript abundance data of the muscle transcriptome of NMRs exposed to hypoxia using the Illumina HiSeq 2500 system to clarify the possible genomic adaptive responses to the hypoxic underground surroundings. The RNA-seq raw FastQ data were mapped against the NMR genome. We identified 2337 differentially expressed genes (DEGs) by comparison of the hypoxic and control groups. Functional annotation of the DEGs by gene ontology (GO) analysis revealed enrichment of hypoxia stress-related GO categories, including ‘biological regulation’, ‘cellular process’, ‘ion transport’ and ‘cell-cell signaling’. Enrichment of DEGs in signaling pathways was analyzed against the Kyoto Encyclopedia of Genes and Genomes (KEGG) database to identify possible interactions between DEGs. The results revealed significant enrichment of DEGs in focal adhesion, the mitogen-activated protein kinase (MAPK) signaling pathway and the glycine, serine and threonine metabolism pathway. Furthermore, inhibition of DEGs (STMN1, MAPK8IP1 and MAPK10) expression induced apoptosis and arrested cell growth in NMR fibroblasts following hypoxia. Thus, this global transcriptome analysis of NMRs can provide an important genetic resource for the study of hypoxia tolerance in mammals. Furthermore, the identified DEGs may provide important molecular targets for biomedical research into therapeutic strategies for stroke and cardiovascular diseases.

## Abstract (precis)

**Summary:** The identified differentially expressed genes in naked mole rat exposed to hypoxia may provide an important genetic resource for further analyses of mammalian tolerance to hypoxia and molecular targets for the prevention of ischemic diseases. This article has an associated First Person interview with the first author of the paper as part of the supplementary information.

## INTRODUCTION

Hypoxia is a serious challenge for aerobic organisms, which depend on aerobic oxidation of glucose for respiration and energy production in the mitochondria (Hayashi, 1968; Savin, 1966). Cerebral ischemia is a common cause of irreparable brain damage (Zhu et al., 2017) and current data show that the incidence of ischemic heart disease is increasing worldwide, especially in middle-income countries (Finegold et al., 2013). Hypoxia plays critical roles in the pathobiology of many diseases, such as cancer, heart failure, myocardial ischemia, stroke, and chronic lung diseases. Humans lose their work capacity at high altitude mainly because of hypoxia, which inhibits the functions of nerves and muscles. Therefore, a better understanding of the mechanisms responsible for hypoxia tolerance in vertebrates is crucial for the prevention and therapy of ischemic diseases, and for developing protective measures against hypoxic environments.

A series of changes in cytology, physiology and molecular biology caused by hypoxic stress leads to disruption of biochemical processes, increased apoptotic level and impaired cell proliferation (Sendoel and Hengartner, 2014). In order to survive in hypoxic environments, cells rapidly sense the hypoxic stress and respond with a series of complex changes, including changes in messenger molecule-regulated signaling and regulatory pathways; activation of some specific transcription factors which are involved in the hypoxia responses, such as the most important oxygen balance regulator in mammals cells, hypoxia inducible factor-1; and regulation of the expression of genes encoding proteins which are required for maintaining homeostasis, cell protection and/or acclimation (Berlow et al., 2017; Mira et al., 2017; Pereira et al., 2014). The changes in many pathways, including hypoxia-inducible pathways and hypoxia-protective pathways resulting from hypoxic stress responses in mammalian cells, play an important role in ion homeostasis, reactive oxygen species scavenging, and cell growth and apoptosis. Mitogen-activated protein kinases (MAPKs) are a class of serine/threonine protein kinases which control cell growth, differentiation, pathological processes, and a variety of important cellular physiological adaptation to environmental stress (e.g. hypoxia). However, various adaptive hypoxia tolerance mechanisms have evolved in different species. Therefore, comprehensive investigations of the transcriptomes of some unique hypoxia-tolerant species are necessary to gain a better understanding of the genetic mechanisms responsible for hypoxia tolerance.

The naked mole rat (NMR; *Heterocephalus glaber*) inhabits subterranean burrow systems, indicating a high tolerance to hypoxia and hypercapnia, thus representing an excellent model for studying mammalian adaptation to life underground and medical applications (Lewis et al., 2016). This species has evolved several physiological characteristics to adapt to hypoxic environment, including a highly developed respiratory system, low basal metabolic rate and high affinity of hemoglobin and myoglobin for oxygen (Gesser et al., 1977; Park et al., 2017). It has been suggested that the injury or pathological changes caused by hypoxia is either attenuated or completely abrogated by this extraordinary level of tolerance to hypoxic environment. However, previous studies have focused exclusively on the ecological function and biochemistry of this species, and knowledge of its hypoxia tolerance mechanisms is limited due to the lack of NMRs genomic data. Next-generation sequencing technologies provide a robust platform for obtaining such data and elucidating the genetic mechanisms underlying hypoxia tolerance in the NMRs (Zhang et al., 2017).

Here, we report the transcriptome of NMR muscle under continuous hypoxic stress. We mapped the reads against the NMR genome and annotated the genes that were differentially expressed in response to hypoxic stress. The results indicated hypoxia-dependent focal adhesion required for cellular communication and activation of the MAPK signaling pathway involved in cell proliferation, apoptosis and metabolism. These findings suggest that gene expression is highly coordinated in NMR muscle in response to hypoxic stress. Furthermore, the identified differentially expressed genes (DEGs) provide an important genetic resource for further analyses of mammalian tolerance to hypoxia and molecular targets for the prevention of ischemic diseases.

## RESULTS

### RNA-seq and differentially expressed genes (DEGs)

To characterize the transcriptomic responses of NMRs to hypoxic stress, 10 cDNA libraries were constructed from both hypoxia-stressed muscle tissue (exposed to 5% O_2_ for 1 h, 4 h, 8 h and 12 h) and control muscle tissue. Two biological replicates were included for each treatment. In total, we generated 493.7 million 125-bp paired clean reads (61.6 Gb of sequence data) through Illumina sequencing after trimming of the adapter sequences and removal of the low-quality sequences (Table S3). To assess the quality of the sequencing data, we mapped the reads generated for this study against the NMR genome using STAR with default options. On average, 93.39% of reads were mapped to the NMR genome, suggesting complete transcript representation in our study (Table S4). We calculated the expression levels of the genes using the DEseq2. DEGs between the samples (1 h versus 0 h, 4 h versus 0 h, 8 h versus 0 h, 12 h versus 0 h) were identified (Tables S5). A total of 2337 DEGs (upregulated, downregulated, and total) were identified and are listed in Table 1. Among the DEGs, a greater number were upregulated at 8 h or 12 h compared with that at 1 h or 4 h.

**Table 1.**
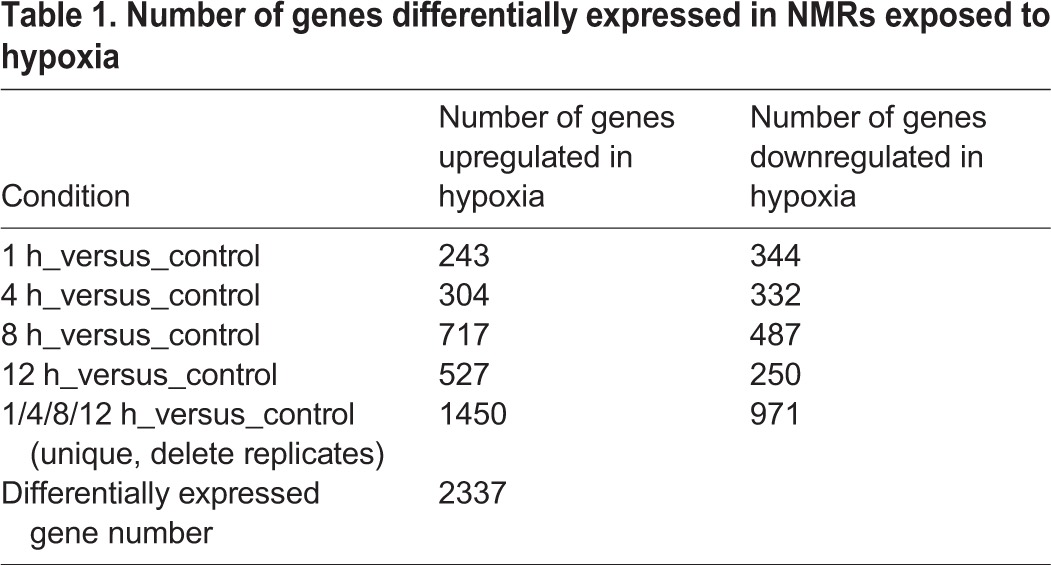
**Number of genes differentially expressed in NMRs exposed to hypoxia**

### Functional categories analysis of DEGs

**Fig. 1.**
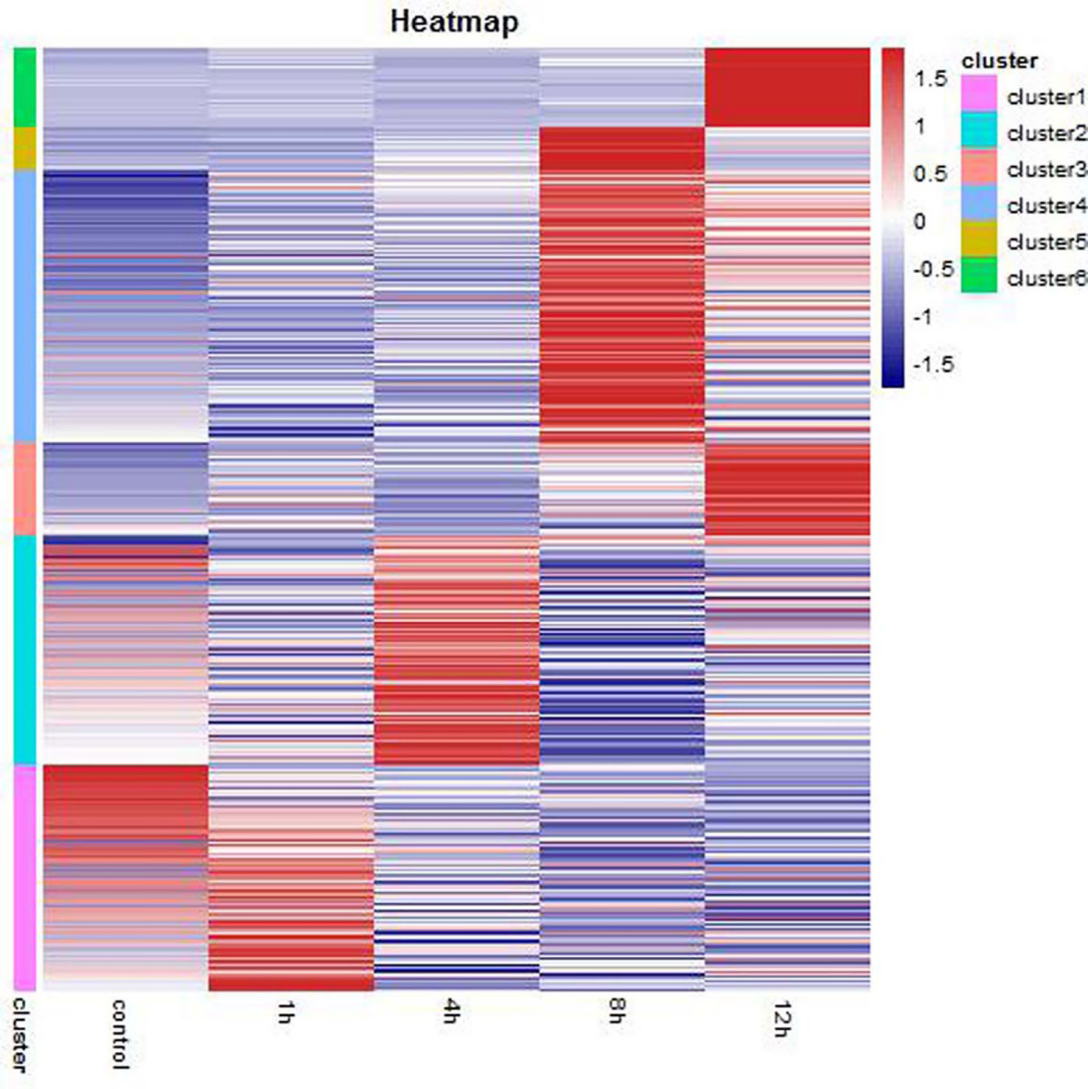
**Hierarchical cluster analysis of DEGs under hypoxic treatment in NMRs.**

A heat map was generated for visualization of the expression patterns of the 2337 identified DEGs (Fig. 1). These DEGs were divided into six clusters through cluster analysis. The number and details of the DEGs in each cluster are listed in Table 2 and Table S6, respectively. In cluster 1, the DEGs were significantly clustered in the 1 h group, while the DEGs in cluster 2 were significantly clustered in the 4 h group. The DEGs in cluster 4 and 5 were significantly clustered in the 8 h group and the DEGs in cluster 3 and cluster 6 were significantly clustered in the 12 h group.

**Table 2.**
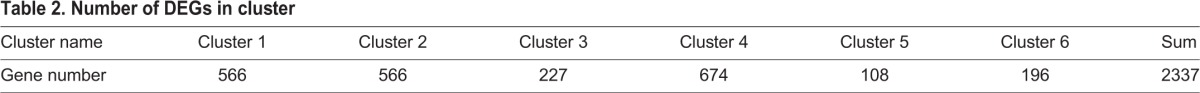
**Number of DEGs in cluster**

To elucidate the functions of the DEGs in each cluster, an overview of the results was obtained using Gene Ontology (GO) Annotation Plot. The GO terms enriched in each cluster are shown in Table S7. In this study, we focused on the DEGs in the main group of biological process. GO analysis indicated that DEGs were enriched in terms related to biological regulation, cell adhesion, cellular process, cellular component organization or biogenesis, nervous system development, metabolic process, ion transport, ion transmembrane transport, cell-cell signaling and synaptic transmission. Another study on rats has revealed enhanced expression of cell adhesion molecules, wound healing and repair bioprocesses in tolerant males exposure to acute hypobaric hypoxia (Sharma et al., 2015). However, cell adhesion has not been reported before as being enriched in NMRs under hypoxic conditions.

On the one hand, cell adhesion is the process by which cells interact and attach to a surface, substrate or another cell, and is mediated by interactions between molecules of the cell surface. On the other hand, contact inhibition is a key anticancer mechanism that arrests cell division at high density. Previous studies have shown that, in cell culture, NMR fibroblast growth is arrested at a much lower density than mouse fibroblasts, demonstrating that NMR fibroblasts display hypersensitivity to contact inhibition, a phenomenon termed ‘early contact inhibition’. In this study, we identified DEGS enriched in cell adhesion and biological adhesion in NMRs after exposure to hypoxia for 1 h. This indicates that NMR cells activate an early contact inhibition mechanism to arrest cell division at an early stage during hypoxia.

### Kyoto encyclopedia of genes and genomes (KEGG) signaling pathway analysis of DEGs

To further elucidate the interaction between the DEGs in each cluster, KEGG signaling pathway analysis was performed. The threshold for significance of each pathway was set at *P*<0.05. The KEGG signaling pathways enriched in each cluster are shown in Table S8. Our analysis indicated that DEGs were enriched in signaling pathways related to focal adhesion, MAPK signaling pathway, dilated cardiomyopathy, circadian rhythm-mammal, cell cycle, and glycine, serine and threonine metabolism and metabolic pathways, which is consistent with the results of the GO analysis.

### Validation of DEGs in muscle tissue

**Fig. 2.**
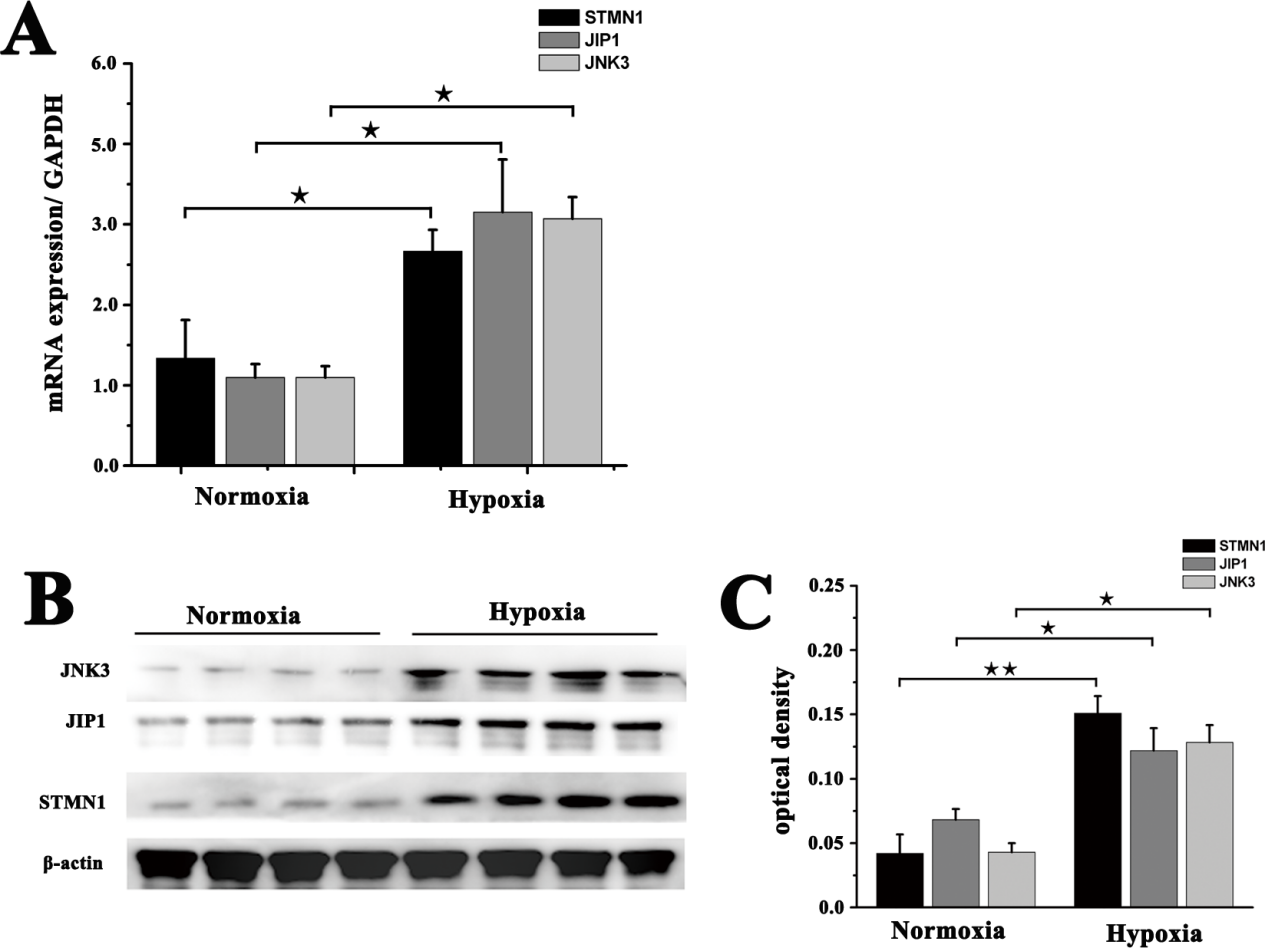
**qRT-PCR and western blot verification of three selected DEGs.** (A) qRT-PCR analysis of STMN1, JIP1 and JNK3 mRNA expression in muscle from NMRs under normoxic or hypoxic (5% O_2_) condition. GAPDH was used as an internal reference. (B) STMN1, JIP1 and JNK3 protein expression was detected by western blot. β-Actin was used as an internal loading control. (C) Band density analysis of STMN1, JIP1 and JNK3 protein expression. The data represent means±s.e.m. of triplicate measurements. ^★^*P*<0.05, ^★★^*P*<0.01 (*t*-test).

To validate the Illumina-Solexa sequencing results, three candidate DEGs in cluster 6 that were associated with the hypoxia response were selected for analysis of expression in muscle tissue by real-time PCR and western blot. These candidates comprised one encoding a microtubule-regulating protein (STMN1), one encoding an enzyme [MAPK8IP1(JIP1)] and one encoding a MAPK protein [MAPK10 (JNK3)]. Although the fold change in their expression detected by sequencing, real-time PCR and western blot did not match exactly, the expression patterns determined for all three genes were consistent, confirming the reliability of the RNA-seq results (Fig. 2).

### Hypoxia induces upregulation of STMN1, MAPK8IP1 and MAPK10 in NMR muscle fibroblasts

**Fig. 3.**
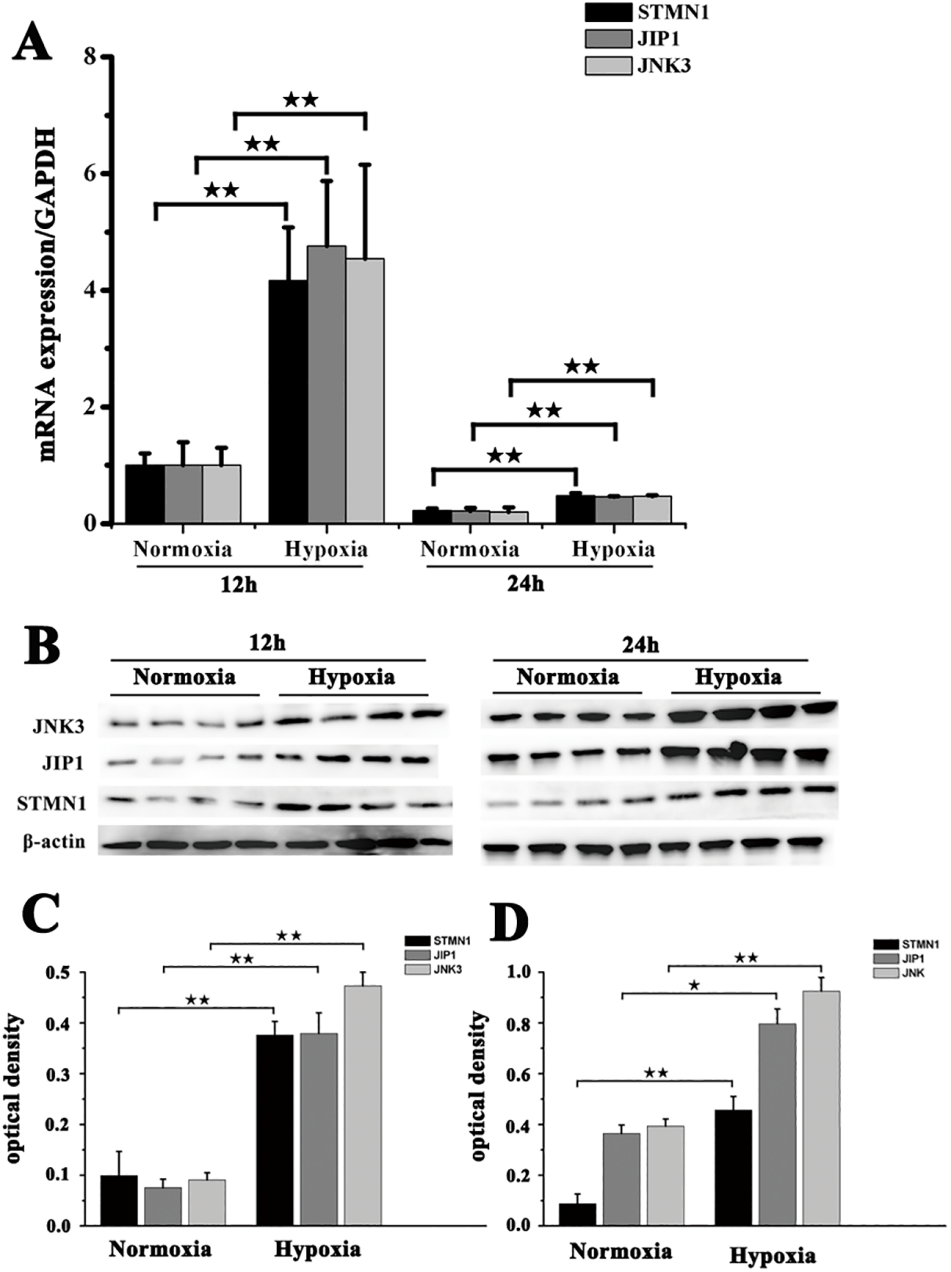
**Expression of STMN1, JIP1 and JNK3 in muscle fibroblasts from NMRs before and after exposure to hypoxia.** (A) Real-time PCR analysis of STMN1, JIP1 and JNK3 mRNA expression in muscle fibroblasts before and after exposure to hypoxia (5% O_2_) for 12 h or 24 h. (B) Western blot detection of STMN1, JIP1 and JNK3 protein expression in fibroblasts before and after exposure to hypoxia (5% O_2_) for 12 h or 24 h. β-actin was used as an internal loading control. (C,D) Band density analysis of STMN1, JIP1 and JNK3 protein expression in fibroblasts before and after exposure to hypoxia (5% O_2_) for (C) 12 h or (D) 24 h. Data represent the mean±s.e.m. of five independent experiments. ^★^*P*<0.05, ^★★^*P*<0.01 (*t*-test).

STMN1, MAPK8IP1 and MAPK10 expression levels in fibroblasts exposed to hypoxia (5% O_2_) for 12 h and 24 h were detected by real-time PCR and western blot. Compared with the control group (normoxia group), fibroblast expression of STMN1, MAPK8IP1 and MAPK10 was significantly upregulated following exposure to hypoxia (Fig. 3). This suggested that hypoxia induced the expression of STMN1, MAPK8IP1 and MAPK10 in fibroblasts.

### siRNA-mediated inhibition of STMN1, MAPK8IP1 and MAPK10 expression

**Fig. 4.**
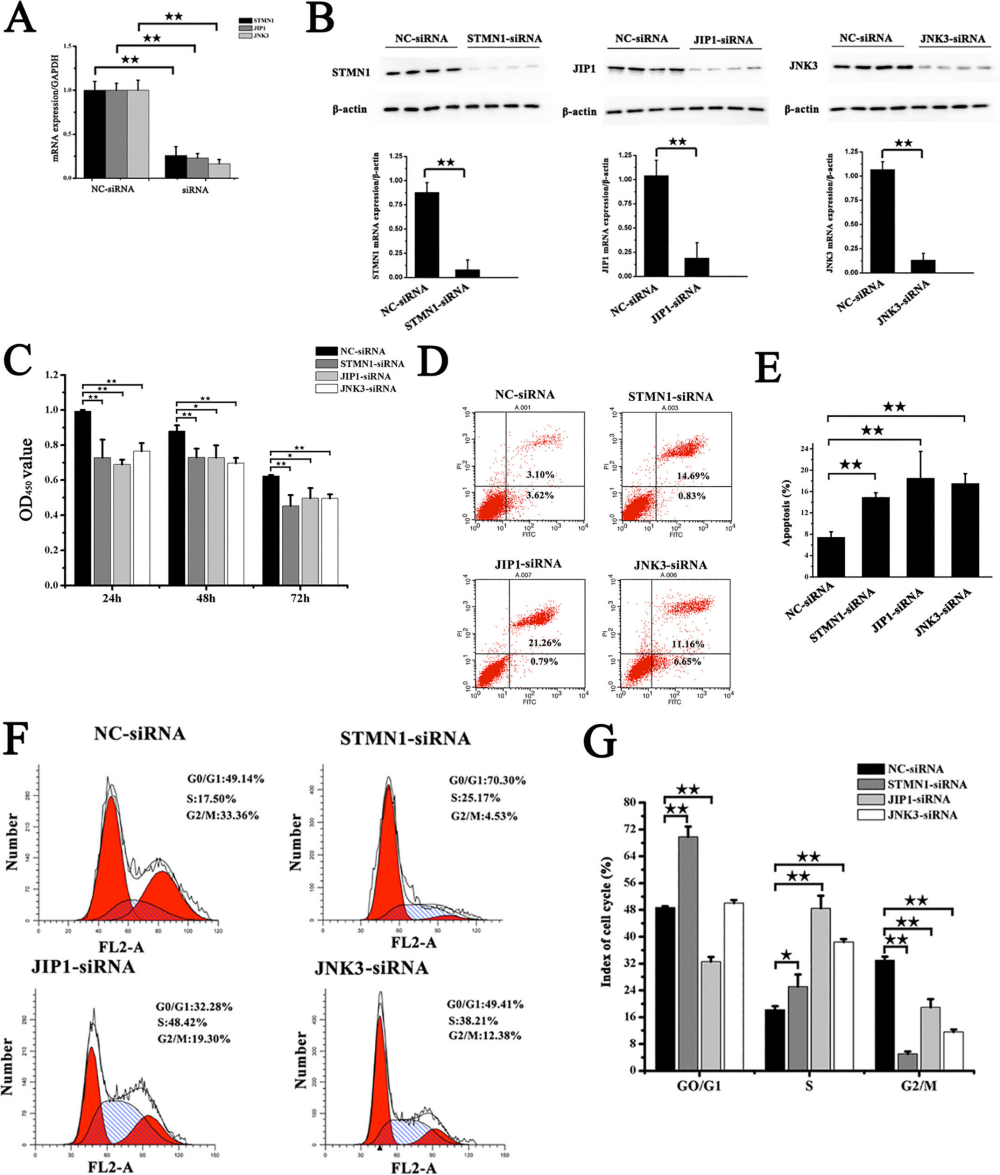
**SiRNA-mediated inhibition of STMN1, JIP1 and JNK3 expression and its effects on cell proliferation, apoptosis and cell cycle progression in NMR muscle fibroblasts.** (A) Real-time PCR analysis of STMN1, JIP1 and JNK3 mRNA levels in fibroblasts transfected with siRNA for 24 h under hypoxic conditions. GAPDH was used as an internal reference. (B) Detection of STMN1, JIP1 and JNK3 protein in fibroblasts after transfection with siRNA for 48 h under hypoxic conditions. (C) Effects of inhibition of STMN1, JIP1 and JNK3 for 24 h, 48 h, or 72 h on the growth of NMR fibroblasts. (D) Effect of inhibition of STMN1, JIP1 and JNK3 for 48 h on cell apoptosis. (E) Statistical analysis of NMR fibroblast apoptosis after transfection with siRNA for 48 h. (F) Flow cytometric (FCM) analysis of the cell cycle distribution of NMR fibroblasts after transfection with siRNA for 48 h. (G) Quantification of FCM results in the different groups. ^★^*P*<0.05, ^★★^*P*<0.01 (*t*-test). Data in A, C E and G are represented as mean±s.e.m.

Expression of STMN1, MAPK8IP1 and MAPK10 mRNA and protein was analyzed by real-time PCR and western blotting to evaluate the efficiency of siRNA-mediated silencing of these genes in transfected fibroblasts. Compared with the control group (NC-siRNA), STMN1, MAPK8IP1 and MAPK10 mRNA levels decreased 75%, 77%, and 84%, respectively, after transfection with siRNA sequences for 24 h (*P*<0.05). These effects were confirmed at the protein level by the western blot after transfection for 48 h (Fig. 4).

### Effect of inhibition of STMN1, MAPK8IP1 and MAPK10 expression on fibroblast proliferation, apoptosis and cell cycle

To determine the effect of STMN1, MAPK8IP1 and MAPK10 inhibition on fibroblast proliferation, a cell proliferation was performed. There were significantly fewer cells following in siRNA-mediated silencing of gene expression (STMN1-siRNA, MAPK8IP1-siRNA and MAPK10-siRNA) compared with those in the control group (Fig. 4C). Annexin-V-FITC staining showed a significant increase in the frequency of apoptotic fibroblasts following siRNA transfection compared with the basal levels detected in the control group (Fig. 4D,E). These results demonstrated that siRNA-mediated silencing of STMN1, MAPK8IP1 and MAPK10 disrupted the cellular mechanisms that regulate apoptosis process. Cell cycle distribution analysis shown in Fig. 4F,G showed that NMR fibroblasts treated with STMN1-siRNA exhibited a significantly greater proportion of cells reaching the GO/G1 and S stages compared with those in the NC-siRNA group (*P*<0.05), while both MAPK8IP1-siRNA and MAPK10-siRNA induced a significantly greater proportion of cells reaching the S stages. These observations confirmed that cell growth was arrested by siRNA.

## DISCUSSION

The brain utilizes more than 20% of the consumed oxygen to generate adenosine triphosphate (ATP) to facilitate the membrane potentials required for synaptic activity, energy utilization and supply (Herculano-Houzel, 2011; Nagase et al., 2014). Neuronal death is thought to be caused by metabolic derangements, including the disruption in oxygen demand and supply, along with the resulting energy deficit through pathogenesis of hypoxic neuronal injury. The premise that suitable hypoxia-tolerant animal model brains can offer vital strategies for developing new applicable therapies to prevent or treat tissue hypoxia injury because of their neurons mounting a wide range of resistance to tissue hypoxia is supported by some evidence. In this study, we report the transcriptome of NMR muscle under continuous hypoxic stress to elucidate the genetic mechanisms underlying hypoxia tolerance in the NMR. A total of 2337 DEGs (upregulated, downregulated, and total) were identified. Among the DEGs, a greater number were upregulated at 8 h or 12 h compared with that at 1 h or 4 h, suggesting differences in the expression patterns at these points during the hypoxic treatment. It can, therefore, be speculated that NMR experienced hypoxic shock (1 h and 4 h) followed by hypoxic stress (8 h and 12 h). Hypoxic shock caused by exposure to 5% O_2_ may result in a shortage of oxygen, leading to strong and rapid changes in the expression of genes involved in sensing oxygen tension and slight and earlier changes in expression of hypoxic-response genes. In contrast, more long-term hypoxic stress causes relatively steady changes in the expression of genes related to metabolism and dramatic changes in the expression of genes involved in cell survival. Hence, in the next studies we focused on the DEGs that were significantly upregulated at 12 h to investigate the exact mechanism underlying the response to hypoxia in NMRs.

We used the Gene Ontology (GO) and Kyoto Encyclopedia of Genes and Genomes (KEGG) databases to perform the functional annotation. The annotation of individual genes was performed using GO terms and genes that can be grouped into networks were analyzed using the KEGG pathways database to provide a functional understanding of genes that work together in a pathway. The biological pathways of model and non-model organisms can be showed through plotting KEGG pathway maps. Only pathways having relevant biological implications being used in the evaluation of transcriptome data are ensured by the identification of significantly enriched pathways through statistical analyses. Thus, we can use genes that rank highly in a pathway identified as being significantly enriched as potential candidates to test the validity of the pathway in functional studies using relevant systems such as knock-out models. In this study, GO analysis indicated that DEGs were enriched in terms related to biological regulation, cell adhesion, cellular process, cellular component organization or biogenesis, nervous system development, metabolic process, ion transport, ion transmembrane transport, cell-cell signaling and synaptic transmission. Cell adhesion has not been reported before as being enriched in NMRs under hypoxic conditions. Most of these GO terms are likely to be associated with hypoxia tolerance. For instance, since the growth of the organism and maintaining homeostasis are controlled through biological regulation including nervous regulation, humoral regulation and nerve-humoral regulation, we assume that the upregulated genes involved in biological regulation maintain homeostasis to avoid disturbances in the acid-base and electrolyte balance under hypoxic conditions. At the same time, other enriched GO terms were ion transport and ion transmembrane transport, suggesting the involvement of activated transmembrane ion transport in maintaining homeostasis. The KEGG pathway analysis showed significant enrichment of MAPK signaling pathway in NMRs exposed to 5% O_2_ for 12 h. Further analysis was then conducted to decipher the pathway network of genes involved in MAPK signaling expressed in response to acute hypoxic stimulation in NMRs. Detection of increased levels of the STMN1, MAPK8IP1 and MAPK10 protein in NMR fibroblasts 12 h and 24 h post-exposure to hypoxic stress consolidates the view that differential expression of MAPK signaling genes identified in NMRs in this study was induced by hypoxia. Hence, our pathway analysis suggests the involvement of MAPK signaling in cell survival under prolonged conditions of acute hypoxic stimulation in NMRs. Overall this study highlights the power of pathway-based analyses for the identification of the most important genes expressed in a transcriptome in response to stimuli. Furthermore, in contrast to time-consuming and laborious non-systematic approaches, our study also shows that pathway-based analyses for the identification of molecular networks of genes expressed in a transcriptome provides a contextual understanding of biological processes induced by hypoxic exposure.

MAPKs are conserved across species from yeast to humans, control cell growth, differentiation, and a variety of important cellular physiological adaptations to environmental stress, inflammatory response and pathological processes. In the present study, STMN1, MAPK8IP1 and MAPK10 were upregulated, suggesting that NMRs respond to prolonged hypoxic stress by activating the MAPK signaling pathway. STMN1 is a microtubule-destabilizing phosphoprotein that is considered to play a crucial role in regulating cellular microtubule dynamics and controlling mitosis (Rubin and Atweh, 2004). Previous studies have showed that STMN1 is highly expressed in many human malignancies and is related to development, invasion and metastasis of tumors as well as being upregulated in normally proliferating cell lines (Rana et al., 2008). In this study, we found that STMN1 was significantly elevated in NMR fibroblasts after exposure to hypoxia for 12 h or 24 h. Furthermore, siRNA-mediated knockdown of STMN1 inhibited cell proliferation and induced apoptosis. This is consistent with the observations reported by Yoshie et al. (2009) who showed that endometrial stathmin is linked to HIF-1 protein accumulation and upregulated VEGF expression via the PI3K/Akt signaling pathway, and may be involved in regeneration of the endometrium during the menstrual cycle in human uterine cells. In addition, inhibition of stathmin expression leads to accumulation of cells in the GO/G1 and S phases of the cell cycle, and is associated with severe mitotic spindle abnormalities and difficulty in the exit from mitosis in NMRs fibroblasts under hypoxic environment. In contrast, interference with stathmin function by forced expression or inhibition of expression in other mammals results in reduced cellular proliferation and accumulation of cells in the G2/M phases (Rubin and Atweh, 2004). Thus, stathmin is critically important not only for the regulation of the function of the mitotic spindle in the later stages of mitosis and for the timely exit from mitosis, but also for the formation of a normal mitotic spindle on entry into mitosis. In combination, these observations indicate that cell proliferation in NMRs is regulated through upregulated STMN1 expression under hypoxic conditions. However, detailed studies using knock-in systems are required to elucidate the mechanisms that control the expression of the individual genes in the MAPK signaling pathway that was identified in this study.

Apart from the significance of upregulation of STMN1 expression, MAPK8IP1 (JIP1) and MAPK10 (JNK3) were identified as other key genes that were upregulated significantly in NMRs subjected to hypoxic stress for 12 h. JNK is a major signal transducer driving the physiological responses to cellular stressors, including ultraviolet (UV)-radiation, genotoxic damage, oxidative stress, endoplasmic reticulum stress, long-chain saturated fatty acids, inflammatory cytokines, and microbial by-products. The outcomes of these responses range from cell death to cell proliferation and survival, depending on the specific context, magnitude, and duration of its activation (Derijard et al., 1994; Kyriakis et al., 1994). JIP-1 has been identified as a cytoplasmic inhibitor of JNK that retains JNK in the cytoplasm, thereby inhibiting JNK-regulated gene expression. Indeed, JNK was identified as a UV-responsive protein kinase mediating the phosphorylation and activation of the cJun transcription factor in response to a variety of stressors (Hibi et al., 1993). The AP-1 transcription factors, which are regulated by JNK, have been linked to cell survival and cell cycle progression, but also to programmed cell death, although evidence indicates that cJun functions mainly a positive regulator of cell proliferation (Shaulian and Karin, 2001, 2002; Thevananther et al., 2004). Indeed, the JNK-cJun pathway was shown to be an early event driving proliferation during liver regeneration by a mechanism involving increased cyclin D1 expression (Alcorn et al., 1990; Schwabe et al., 2003; Stepniak et al., 2006). JNK isoforms (JNK1 and JNK2) are reported to be expressed ubiquitously, while JNK3 expression is restricted to the brain, testis, and pancreatic β-cells (Solinas and Karin, 2010); however, our study showed that JNK3 expression was induced in NMR muscle by hypoxia. Given that transient moderate JNK activation may promote stress tolerance, whereas for sustained JNK activation the expected outcome is cell death (Chen et al., 1996; Solinas and Becattini, 2017), it is likely that the upregulation of JIP1 in response to hypoxia observed in our study could lead to moderate JNK activation, which promotes cell survival, and therefore could account for the expression of JNK3 and JIP1 detected in this study. Furthermore, inhibition of JIP1 expression led to a significant increase in NMR fibroblast apoptosis. This is consistent with the report that maximal JNK activation is observed during the initiation of apoptosis when JIP1 is intact, whereas cleavage of JIP1 correlates with JNK inactivation and progression of apoptosis in HeLa cells (Vaishnav et al., 2011). Overall, the repertoire of genes clustered in the MAPK signaling pathway identified in this study is consistent with the genes induced by hypoxic stress in higher vertebrate cells, suggesting that the mechanisms that promote fibroblast survival in response to hypoxia in NMRs are similar to those of other vertebrates.

### Conclusions

In the present study, we explored the transcriptomic changes of NMRs muscle in response to hypoxic stress over time and identified numerous DEGs with primary involvement in cell adhesion, cell-cell signaling, and metabolism. Furthermore, we found that the temporal expression pattern of the DEGs varied greatly according to the length of time that NMRs were exposed to hypoxic stress. We also identified a greater number of upregulated DEGs at 8 h or 12 h compared with those at 1 h or 4 h. These findings suggest that gene expression in NMR muscle is highly coordinated in response to hypoxic shock at 1 h or 4 h and hypoxic stress at 8 h or 12 h. Furthermore, a contextual understanding of the biological relevance of DEGs expressed in a transcriptome can be obtained through pathway-based analysis. It can be predicted that data presented here will expand our understanding of the cellular mechanisms underlying the response of NMR muscle to hypoxic stress.

## MATERIALS AND METHODS

### Animals and hypoxic conditions

Adult NMRs were purchased from the Department of Zoology at the University of Cape Town and housed at the Laboratory Animal Center of the Second Military Medical University. The NMRs were kept under normoxia for 14 days prior to hypoxic exposure. To test for acute hypoxic stress, animals with a similar weight (40–60 g) were placed in a chamber (dimensions 81×58×121 cm) divided into three compartments. Adult NMRs (aged 8 months) were subjected to acute hypoxic conditions (5% O_2_) by keeping them in the chamber for 1 h, 4 h, 8 h and 12 h; control groups were subjected to normoxic conditions (21% O_2_). Two biological replicates (from two animals) were analyzed for each treatment. All animal protocols were approved by the Institutional Ethics Committee and were consistent with current regulations [GB14925-2001: Laboratory Animal Requirements of Environment and Housing Facilities (Chinese version)].

### Sample collection

After hypoxic exposure for 1 h, 4 h, 8 h, and 12 h, animals were euthanized by intraperitoneally injecting a lethal dose (150 mg/kg) of pentobarbital sodium obtained from Sigma–Aldrich Co. (St Louis, MO, USA). The normoxic group also received intraperitoneal injection of an overdose of pentobarbital. Muscle tissues were immediately removed and frozen in liquid nitrogen until further use.

### Extraction of total RNA and sequencing

For transcriptome sequencing, total RNA was extracted from muscle tissue samples obtained from animals exposed to hypoxia (5% O_2_) for 1 h, 4 h, 8 h and 12 h and corresponding controls (two biological replicates per treatment) using TRIzol reagent (Invitrogen, Life Technologies Corp., Carlsbad, CA, USA) according to the instructions. The purity and quality of total RNA was then evaluated spectrophotometrically (A_260_/A_280_). Ten cDNA libraries were constructed and sequenced by WuXi Genome Center (Shanghai, China). Using standardized procedures and monitoring with a standard Quality Control System, RNAs with poly(A) tails were purified from total RNA using oligonucleotide (dT) magnetic beads and then fragmented into short sequences that were used for cDNA synthesis. For each sample, an RNA sequencing library was constructed using the Illumina mRNA-Seq Prep Kit (Illumina Inc., San Diego, CA, USA). Paired-end RNA-seq libraries were prepared following Illumina's protocols and sequenced on the Illumina HiSeq 2500 sequencing platform with a 125-bp read length following end repair, adapter ligation and PCR amplification. Image output data from the sequencer were transformed into raw sequence data by base calling and stored in FastQ format.

### Gene expression and functional analysis of DEGs

We downloaded the genome file and gene annotation of NMRs from UCSC (https://genome.ucsc.edu). The RNA-seq raw FastQ data was mapped against the NMR genome using STAR with default options (STAR: ultrafast universal RNA-seq aligner). We detected DEGs between the hypoxia-stressed and control groups by DEseq2 (moderated estimation of fold change and dispersion for RNA-seq data with DESeq2), and also checked for their biological function in DAVID (https://david.ncifcrf.gov).

### Validation of expression of genes involved in MAPK signaling pathway by real-time PCR

To validate the reliability of the RNA-seq results, the expression of three candidate DEGs in response to hypoxic stress was measured by real-time PCR. A total of 500 ng total RNA was converted into single-stranded cDNA using TIANScript cDNA First-Strand Kit (Tiangen Biochemical Technology Co., Ltd., Beijing, China). The cDNA templates were then diluted fivefold prior to use. Gene expression (mRNA levels) was measured using the StepOnePlus Real-time PCR Detection System (Applied Biosystems, Warrington, UK) with SYBR® Green Master Mix (Applied Biosystems) and the following conditions: 30 s at 95°C; followed by 40 cycles of 95°C for 5 s, 60°C for 34 s and 72°C for 30 s; melting curve stage: 95°C for 15 s, 60°C for 1 min and 95°C for 15 s. Specific primers were designed using Primers 5.0 software and the sequences are listed in Table S1. The specificity of each PCR product was confirmed by melting curve analysis and agarose gel analysis. The expression of each gene was analyzed using three biological replicates. Relative gene expression levels were normalized against expression of the internal reference gene glyceraldehyde-3-phosphate dehydrogenase (GAPDH) and were calculated using the ΔΔCt method.

### Western blot analysis

Total proteins were extracted from muscle tissue using RIPA lysis buffer containing 1 mM phenylmethylsulfonyl fluoride (PMSF) (Wuhan Boster Biotech, Wuhan, China). Proteins were then separated by polyacrylamide gel electrophoresis (90 V for 30 min then 120 V for 60 min), and transferred to a Polyvinylidene Fluoride (PVDF) membrane (Bio-Rad Mini Protean 3 gel system, Millipore Immobilon P^SQ^) (300 mA, 90 min). Membranes were then blocked by incubation with 5% skimmed milk overnight at 4°C, followed by incubation at 4°C for 2 h with primary antibodies for the detection of STMN1 (Proteintech, Chicago, USA), MAPK8IP1 (Cell Signaling Technology, Boston, USA) and MAPK10 (Abcam, Cambridge, UK) at a dilution of 1:1000. Subsequently, the membranes were washed three times with Tris-buffered saline with Tween 20 (TBST) (5 min per wash) and incubated with a goat anti-rabbit IgG antibodies diluted 1:4000 in blotting buffer (5% skimned milk). Blots were stripped with stripping buffer and reprobed with anti-β-actin antibody (Wuhan Boster Biotech, Wuhan, China) at a dilution of 1:2000 to confirm equal protein loading among the samples. Finally, immunoreactive bands were visualized using the Kodak Gel Logic 4000 R Imaging System (Carestream, USA).

### Cell culture and hypoxic stress

Fibroblasts were isolated from NMR muscle tissue according to previously reported methods (Goetsch et al., 2015). Primary fibroblasts were cultured at 37°C under 5% CO_2_ and 21% O_2_ in complete Dulbecco's Modified Eagle's medium (DMEM; supplemented with 15% fetal bovine serum, 100 units/ml penicillin and streptomycin). Fibroblasts were subjected to hypoxic stress by exposure to 5% O_2_, 5% CO_2_ and 90% N_2_. All cell lines were used at an early passage (5–15 population doublings).

### Small interfering RNA-mediated inhibition of gene expression

Small interfering RNA (siRNA) was synthesized by Biomics Biotechnology Co., Ltd. (Nantong, China). The siRNA sequences are listed in Table S2. Fibroblasts in complete DMEM were seeded in six-well plates (2×10^5^cells) and cultured at 37°C under 5% O_2_, 5% CO_2_ and 90% N_2_. At 70–80% confluence, cells were incubated for 6 h in complete medium free of penicillin and streptomycin before transfection for a further 6 h with 50 nM siRNA using lipofectamine 2000 (lipo2000, Invitrogen, Carlsbad, CA, USA) according to the manufacturer's instructions. Subsequently, the fibroblasts were cultured in complete DMEM. The efficiency of siRNA-mediated silencing was confirmed by real-time PCR analysis of mRNA expression levels of target genes (STMN1, JIP1, and JNK3) after transfection for 24 h and western blot analysis of protein expression levels after 48 h.

### Cell proliferation assay

Cell proliferation was measured using Cell Proliferation and Cytotoxicity Assay Kits (Signalway Antibody, USA) according to the manufacturer's instructions. Fibroblasts were seeded in a 96-well flat-bottomed plate (1×10^4^ cells/well) and incubated for up to three days after transfection with siRNA. Three replicates were assessed for each treatment. Viable cells were counted at 24, 48, and 72 h after treatment by the addition of 10 μl of the Cell Proliferation and Cytotoxicity Assay Kit solution to each well and incubation for 2 h in a humidified incubator at 37°C under 5% CO_2_. Absorbance was measured using a microplate reader (Synergy HT, Bio-Tek) at 450 nm. Proliferation was calculated as the percentage of surviving cells in each treated group relative to control group. Data analysis was based on the average of three independent experiments.

### Analysis of apoptotic level

Fibroblasts (1×10^5^ cells/ml) were cultured in six-well plates to 50%–60% confluence 5% O_2_, 5% CO_2_ and 90% before transfection with siRNA for 48 h. Subsequently, cells were released by trysinization, washed twice with ice-cold PBS and resuspended with 300 μl buffer containing 5 μl annexin V-fluorescein isothiocyanate and 5 μl propidium iodide (PI; Nanjing KGI biotechnology, China). Cells were incubated in the dark at 37°C for 30 min before apoptotic rates were analyzed by flow cytometry (Becton Dickinson, USA) and ModFit LT software.

### Cell cycle analysis

For cell cycle analysis, fibroblasts were harvested and fixed with ice-cold 75% ethanol at 4°C for at least 6 h. Fixed fibroblasts were then resuspended in PBS buffer containing PI and RNaseA at room temperature for 30 min in the dark. The nuclear DNA content and the percentage of cells in the S+G2 phase were analyzed by flow cytometry.

### Statistical analysis

Data were presented as mean±s.d. We used SPSS17.0 statistical software for statistical analysis of the data. We assessed the statistical significance of the differences between the means using *t*-tests. *P*-values of <0.05 were considered to indicate statistical significance.

## Supplementary Material

Supplementary information

First Person interview

## References

[BIO028548C1] Alcorn J. A., Feitelberg S. P. and Brenner D. A. (1990). Transient induction of c-jun during hepatic regeneration. *Hepatology* 11, 909-915. 10.1002/hep.1840110602 2114348

[BIO028548C2] Berlow R. B., Dyson H. J. and Wright P. E. (2017). Hypersensitive termination of the hypoxic response by a disordered protein switch. *Nature* 543, 447-451. 10.1038/nature21705 28273070 PMC5375031

[BIO028548C3] Chen Y. R., Wang X., Templeton D., Davis R. J. and Tan T. H. (1996). The role of c-Jun N-terminal kinase (JNK) in apoptosis induced by ultraviolet C and gamma radiation. Duration of JNK activation may determine cell death and proliferation. *J. Biol. Chem.* 271, 31929-31936. 10.1074/jbc.271.50.31929 8943238

[BIO028548C4] Dérijard B., Hibi M., Wu I.-H., Barrett T., Su B., Deng T., Karin M. and Davis R. J. (1994). JNK1: a protein kinase stimulated by UV light and Ha-Ras that binds and phosphorylates the c-Jun activation domain. *Cell* 76, 1025-1037. 10.1016/0092-8674(94)90380-8 8137421

[BIO028548C5] Finegold J. A., Asaria P. and Francis D. P. (2013). Mortality from ischaemic heart disease by country, region, and age: statistics from World Health Organisation and United Nations. *Int. J. Cardiol.* 168, 934-945. 10.1016/j.ijcard.2012.10.046 23218570 PMC3819990

[BIO028548C6] Gesser H., Johansen K. and Maloiy G. M. O. (1977). Tissue metabolism and enzyme activities in the rodent Heterocephalus glaber, a poor temperature regulator. *Comp. Biochem. Physiol. B* 57, 293-296. 10.1016/0305-0491(77)90056-6 233774

[BIO028548C7] Goetsch K. P., Snyman C., Myburgh K. H. and Niesler C. U. (2015). Simultaneous isolation of enriched myoblasts and fibroblasts for migration analysis within a novel co-culture assay. *BioTechniques* 58, 25-32. 10.2144/000114246 25605577

[BIO028548C8] Hayashi T. (1968). [Carbohydrate metabolism and ECG changes in hypoxia stress]. *Munch. Med. Wochenschr.* 110, 1768-1776. 5755401

[BIO028548C9] Herculano-Houzel S. (2011). Scaling of brain metabolism with a fixed energy budget per neuron: implications for neuronal activity, plasticity and evolution. *PLoS One* 6, e17514 10.1371/journal.pone.0017514 21390261 PMC3046985

[BIO028548C10] Hibi M., Lin A., Smeal T., Minden A. and Karin M. (1993). Identification of an oncoprotein- and UV-responsive protein kinase that binds and potentiates the c-Jun activation domain. *Genes Dev.* 7, 2135-2148. 10.1101/gad.7.11.2135 8224842

[BIO028548C11] Kyriakis J. M., Banerjee P., Nikolakaki E., Dai T., Rubie E. A., Ahmad M. F., Avruch J. and Woodgett J. R. (1994). The stress-activated protein kinase subfamily of c-Jun kinases. *Nature* 369, 156-160. 10.1038/369156a0 8177321

[BIO028548C12] Lewis K. N., Soifer I., Melamud E., Roy M., McIsaac R. S., Hibbs M. and Buffenstein R. (2016). Unraveling the message: insights into comparative genomics of the naked mole-rat. *Mamm. Genome* 27, 259-278. 10.1007/s00335-016-9648-5 27364349 PMC4935753

[BIO028548C13] Mira M. M., El-Khateeb E. A., SayedAhmed H. I., Hill R. D. and Stasolla C. (2017). Are avoidance and acclimation responses during hypoxic stress modulated by distinct cell-specific mechanisms? *Plant Signal. Behav.* 12, e1273304 10.1080/15592324.2016.1273304 28010170 PMC5289513

[BIO028548C14] Nagase M., Takahashi Y., Watabe A. M., Kubo Y. and Kato F. (2014). On-site energy supply at synapses through monocarboxylate transporters maintains excitatory synaptic transmission. *J. Neurosci.* 34, 2605-2617. 10.1523/JNEUROSCI.4687-12.2014 24523550 PMC6802746

[BIO028548C15] Park T. J., Reznick J., Peterson B. L., Blass G., Omerbašić D., Bennett N. C., Kuich P., Zasada C., Browe B. M., Hamann W. et al. (2017). Fructose-driven glycolysis supports anoxia resistance in the naked mole-rat. *Science* 356, 307-311. 10.1126/science.aab3896 28428423

[BIO028548C16] Pereira E. R., Frudd K., Awad W. and Hendershot L. M. (2014). Endoplasmic reticulum (ER) stress and hypoxia response pathways interact to potentiate hypoxia-inducible factor 1 (HIF-1) transcriptional activity on targets like vascular endothelial growth factor (VEGF). *J. Biol. Chem.* 289, 3352-3364. 10.1074/jbc.M113.507194 24347168 PMC3916539

[BIO028548C17] Rana S., Maples P. B., Senzer N. and Nemunaitis J. (2008). Stathmin 1: a novel therapeutic target for anticancer activity. *Expert Rev. Anticancer Ther.* 8, 1461-1470. 10.1586/14737140.8.9.1461 18759697

[BIO028548C18] Rubin C. I. and Atweh G. F. (2004). The role of stathmin in the regulation of the cell cycle. *J. Cell. Biochem.* 93, 242-250. 10.1002/jcb.20187 15368352

[BIO028548C19] Savin V. M. (1966). Comparative analysis of the effect of overloading and hypoxia on the oxygen tension in the brain tissues. *Biull. Exp. Biol. Med.* 61, 488-492. 10.1007/BF01892434

[BIO028548C20] Schwabe R. F., Bradham C. A., Uehara T., Hatano E., Bennett B. L., Schoonhoven R. and Brenner D. A. (2003). c-Jun-N-terminal kinase drives cyclin D1 expression and proliferation during liver regeneration. *Hepatology* 37, 824-832. 10.1053/jhep.2003.50135 12668975

[BIO028548C21] Sendoel A. and Hengartner M. O. (2014). Apoptotic cell death under hypoxia. *Physiology (Bethesda)* 29, 168-176. 10.1152/physiol.00016.2013 24789981

[BIO028548C22] Sharma P., Bansal A. and Sharma P. C. (2015). RNA-seq-based transcriptome profiling reveals differential gene expression in the lungs of Sprague-Dawley rats during early-phase acute hypobaric hypoxia. *Mol. Genet. Genomics* 290, 2225-2240. 10.1007/s00438-015-1064-0 26050109

[BIO028548C23] Shaulian E. and Karin M. (2001). AP-1 in cell proliferation and survival. *Oncogene* 20, 2390-2400. 10.1038/sj.onc.1204383 11402335

[BIO028548C24] Shaulian E. and Karin M. (2002). AP-1 as a regulator of cell life and death. *Nat. Cell Biol.* 4, E131-E136. 10.1038/ncb0502-e131 11988758

[BIO028548C25] Solinas G. and Becattini B. (2017). JNK at the crossroad of obesity, insulin resistance, and cell stress response. *Mol. Metab.* 6, 174-184. 10.1016/j.molmet.2016.12.001 28180059 PMC5279903

[BIO028548C26] Solinas G. and Karin M. (2010). JNK1 and IKKbeta: molecular links between obesity and metabolic dysfunction. *FASEB J.* 24, 2596-2611. 10.1096/fj.09-151340 20371626

[BIO028548C27] Stepniak E., Ricci R., Eferl R., Sumara G., Sumara I., Rath M., Hui L. and Wagner E. F. (2006). c-Jun/AP-1 controls liver regeneration by repressing p53/p21 and p38 MAPK activity. *Genes Dev.* 20, 2306-2314. 10.1101/gad.390506 16912279 PMC1553212

[BIO028548C28] Thevananther S., Sun H., Li D., Arjunan V., Awad S. S., Wyllie S., Zimmerman T. L., Goss J. A. and Karpen S. J. (2004). Extracellular ATP activates c-jun N-terminal kinase signaling and cell cycle progression in hepatocytes. *Hepatology* 39, 393-402. 10.1002/hep.20075 14767992

[BIO028548C29] Vaishnav M., MacFarlane M. and Dickens M. (2011). Disassembly of the JIP1/JNK molecular scaffold by caspase-3-mediated cleavage of JIP1 during apoptosis. *Exp. Cell Res.* 317, 1028-1039. 10.1016/j.yexcr.2011.01.011 21237154 PMC3063339

[BIO028548C30] Yoshie M., Miyajima E., Kyo S. and Tamura K. (2009). Stathmin, a microtubule regulatory protein, is associated with hypoxia-inducible factor-1alpha levels in human endometrial and endothelial cells. *Endocrinology* 150, 2413-2418. 10.1210/en.2008-1333 19179443

[BIO028548C31] Zhang Y., Huang H., Zhang D., Qiu J., Yang J., Wang K., Zhu L. and Fan J. (2017). A Review on Recent Computational Methods for Predicting Noncoding RNAs. *Biomed. Res. Int.* 2017, 9139504 10.1155/2017/9139504 28553651 PMC5434267

[BIO028548C32] Zhu W., Ye Y., Liu Y., Wang X. R., Shi G. X., Zhang S. and Liu C. Z. (2017). Mechanisms of acupuncture therapy for cerebral ischemia: an evidence-based review of clinical and animal studies on cerebral ischemia. *J. Neuroimmune Pharmacol.* 12, 575-592. 10.1007/s11481-017-9747-4 28527041

