# Supplementary material for: Transcriptome sequencing of the naked mole rat (Heterocephalus glaber) and identification of hypoxia tolerance genes
Source: Biol Open. 2017 Nov 14;6(12):1904–12. doi: 10.1242/bio.028548 (PMC5769652; doi:10.1242/bio.028548)
Supplement: Supplementary information [file biolopen-6-028548-s1.pdf]

© 2017. Published by The Company of Biologists Ltd | Biology Open (2017): doi:10.1242/bio.028548: Supplementary information

FIRST PERSON

First person – Bang Xiao

First Person is a series of interviews with the first authors of a selection of papers published in Biology Open, helping early-career researchers promote themselves alongside their papers. Bang Xiao is first author on 'Transcriptome sequencing of the naked mole rat (*Heterocephalus glaber*) and identification of hypoxia tolerance genes', published in BiO. Bang is a PhD student in the lab of Shufang Cui at The Second Military Medical University, Shanghai, China, investigating molecular mechanisms of hypoxia tolerance in the naked mole rat.

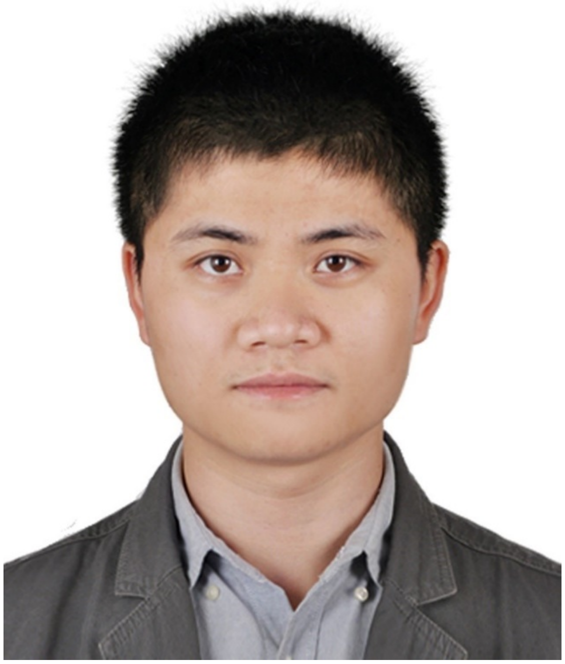

Bang Xiao

What is your scientific background and the general focus of your lab?

I have a BSc in veterinary medicine, and an MSc in zoology. My PhD is in molecular genetics, where under the mentorship of Dr Shuhan Sun, I am studying the mechanisms underlying naked mole rat hypoxia tolerance. I am also investigating the role of H_2O_2 treatment or serum deprivation in autophagy and apoptosis in naked mole rat skin fibroblasts. Furthermore, I am exploring the role of the PI3K/Akt signaling pathway in regulating autophagy and apoptosis of naked mole rat hepatic stellate cells under hypoxic condition. The lab specifically focusses on the mechanism of anti-aging, hypoxia resistance in the naked mole rat.

How would you explain the main findings of your paper to non-scientific family and friends?

Genes that control cell proliferation, apoptosis (cell death) and metabolism are differentially expressed in response to hypoxic stress (low oxygen conditions). The protective roles that these genes play are the same as the emergency departments' roles when a crisis happens. Some signaling pathway involved in cell proliferation and apoptosis are activated when cells are exposed to hypoxia. This is like many departments working together to deal with the crisis. In this paper, we report how naked mole rat muscle genes are expressed under continuous hypoxic stress. We mapped the reads against the naked mole rat genome and annotated the genes that were differentially expressed in response to hypoxic stress.

"We found that the temporal expression pattern of the DEGs varied greatly according to the length of time that naked mole rats were exposed to hypoxic stress."

What are the potential implications of these results for your field of research?

The identified differentially expressed genes in our work provide an important genetic resource for further analyses of mammalian tolerance to hypoxia and molecular targets for the prevention of ischemic diseases.

What has surprised you the most while conducting your research?

The results that indicated hypoxia-dependent focal adhesion is required for cellular communication and activation of the MAPK signaling pathway involved in cell proliferation, apoptosis and metabolism are the most surprising. We explored the transcriptomic changes of naked mole rat muscle in response to hypoxic stress over time and identified numerous differentially expressed genes (DEGs) with primary involvement in cell adhesion, cell-cell signaling, and metabolism. Furthermore, we found that the temporal expression pattern of the DEGs varied greatly according to the length of time that naked mole rats were exposed to hypoxic stress. We also identified a greater number of upregulated DEGs at 8 h or 12 h compared with those at 1 h or 4 h. These findings suggest that gene expression in naked mole rat muscle is highly coordinated in response to hypoxic shock at 1 h or 4 h and hypoxic stress at 8 h or 12 h.

What changes do you think could improve the professional lives of early-career scientists?

More availability to journal articles through open access, earlier and more opportunities to learn relevant experimental techniques. Because the process of learning experimental techniques in which they can well know how the results were obtained can deepen their understanding of the results in the articles. It is often difficult for early career scientists to understand the experimental results and the conclusions in the articles written with a non-native languages.

Bang Xiao's contact details: Laboratory Animal Centre, The Second Military Medical University, Shanghai 200433, China.

E-mail: xiaobangjinli@163.com

Biology Open (2017): doi:10.1242/bio.028548: Supplementary information

What’s next for you?

After I complete my PhD, I will take up a position as a research scientist in the Department of Medical Genetics here at The Second Military Medical University. I will remain in the field of aging research using the natural model of the anti-aging naked mole rat.

Reference

Xiao, B., Li, L., Xu, C., Zhao, S., Lin, L., Cheng, J., Yang, W., Cong, W., Kan, G. and Cui, S. (2017). Transcriptome sequencing of the naked mole rat (*Heterocephalus glaber*) and identification of hypoxia tolerance genes. *Biol. Open* **6**, doi:10.1242/bio.028548.

Biology Open
